# The Purinergic Receptor P2X5 Modulates Glucose Metabolism and Expression of Thermogenic Genes in Brown Adipose Tissue

**DOI:** 10.3390/ijms26136474

**Published:** 2025-07-04

**Authors:** Michelle Y. Jaeckstein, Lisa Miegel, Janina Behrens, Tobias Stähler, Björn-Philipp Diercks, Markus Heine, Friedrich Koch-Nolte, Joerg Heeren

**Affiliations:** 1 Department of Biochemistry and Molecular Cell Biology, University Medical Center Hamburg-Eppendorf, 20246 Hamburg, Germany; m.jaeckstein@uke.de (M.Y.J.); miegel.lisa@gmail.com (L.M.); j.behrens@uke.de (J.B.); b.diercks@uke.de (B.-P.D.); ma.heine@uke.de (M.H.); 2 Institute of Immunology, University Medical Center Hamburg-Eppendorf, 20246 Hamburg, Germany; tobias.staehler@ukbonn.de (T.S.); nolte@uke.de (F.K.-N.)

**Keywords:** brown adipose tissue, purinergic signalling, P2X5, ion channels, glucose metabolism, extracellular ATP

## Abstract

Next to adrenergic signalling, purinergic pathways mediated by extracellular adenine nucleotides have been described to shape thermogenic and metabolic functions in brown adipose tissue (BAT). Here we describe high expression of P2X5 that is activated by ATP in mature adipocytes of BAT and differentiated brown adipocytes in vitro. The levels of other P2X family members were much lower, or expression was restricted to tissue-resident macrophages or endothelial cells. Global and brown adipocyte-specific P2rx5 deficiency resulted in lower expression of the uncoupling protein 1 (UCP1). However, indirect calorimetry studies showed that P2X5 did not affect systemic energy expenditure. Of note, glucose tolerance was impaired under chow and obesogenic high-fat diet conditions, which can be explained by lower glucose disposal into BAT but not into other organs. In summary, these data indicate a modulatory role of P2X5 in systemic and BAT-specific glucose metabolism.

## 1. Introduction

With their central role in energy handling, adipose tissues are essential for maintaining whole-body metabolic homeostasis. Accordingly, dysregulations in adipose tissue depots and excessive nutrient storage drive the development of metabolic diseases and chronic inflammatory processes [1,2]. The incidence of obesity and type 2 diabetes has risen dramatically in recent decades, leading to associated diseases becoming one of the main causes of morbidity and mortality in modern societies. In recent years, the stimulation of energy combustion by brown and white adipose tissues (BAT, WAT) has been suggested as a novel target for combating obesity and associated comorbidities [3,4,5,6]. The main function of thermogenic brown adipocytes within BAT and beige adipocytes present in WAT is to generate heat for body temperature maintenance at cold ambient temperatures. In this process, known as adaptive thermogenesis, activated brown and beige adipocytes internalize and burn large amounts of glucose as well as fatty acids delivered by triglyceride-rich lipoproteins [7,8,9]. In addition, a number of studies suggest that activated BAT modulates energy homeostasis by secreting endocrine factors, thereby orchestrating systemic metabolism of, e.g., the liver, heart, and muscles [10,11,12,13]. Accordingly, due to its potential to burn excess calories and by the secretion of endocrine hormones, BAT activation and/or the prevention of BAT degeneration observed in response to ageing have been proposed as a therapeutic target for the treatment of age- and obesity-associated diseases [3,14,15]. Energy uptake and activation of thermogenic adipocytes is primarily induced by norepinephrine, which is released from sympathetic nerves innervating BAT and WAT. Subsequent beta-adrenergic signalling and heat generation in thermogenic adipocytes is mediated by the uncoupling protein 1 (UCP1) at the inner mitochondrial membrane. By causing a proton leak, UCP1 uncouples the respiratory chain from ATP synthesis and releases the energy stored in the protein gradient as heat. In addition to UCP1-dependent thermogenesis, a number of so-called futile cycles have been identified that contribute to adaptive thermogenesis in heat-producing adipocytes [16].

In the context of the current study, it is of note that mice lacking all beta-adrenergic receptors (β1, β2, β3) are able to adapt to cold conditions and display even higher UCP1 levels in BAT, suggesting additional regulatory pathways that shape thermogenic responses, e.g., purinergic signalling [17]. This hypothesis was supported by the observation that extracellular ATP levels rise in parallel with norepinephrine in response to BAT stimulation [18]. This can be explained by co-secretion of ATP and norepinephrine, which are stored in the same vesicles of sympathetic neurons [17,19,20]. However, the relevance of ATP as a purinergic signal sensed by thermogenic adipocytes has not been fully elucidated. As a ligand of the purinergic receptors of the P2X family, extracellular ATP can shape ion channel-mediated signalling pathways [21,22]. With this ability to regulate cytosolic ion concentrations, e.g., Ca^2+^, and downstream signalling cascades, P2X receptors are responsible for the activation of various cellular processes [23,24,25], and thus possibly also thermogenic responses in brown or beige adipocytes. Recently, we showed that P2X7 is dispensable for energy expenditure and metabolic homeostasis in response to cold exposure [26]. However, P2X7 is mainly expressed by myeloid cells in WAT and BAT, which may explain the negative outcome. Of note, one member of the P2X family, namely the ATP receptor P2X5, has been shown to be highly expressed in BAT compared to various other tissues, serving as a novel marker for brown adipocytes [27]. For its ion channel function it is known that P2X5 can form functional homo- and heterotrimers by assembling with other P2X subunits such as P2X1 and P2X6 [24,28,29]. However, the relevance of P2X5 expressed by brown adipocytes for adaptive thermogenesis and its potential metabolic function have not been studied so far.

In the current study, we confirmed the high expression of P2X5 primarily by brown adipocytes in mice and its induction upon adipogenic differentiation. To further assess the relevance of P2X5 in thermogenesis and metabolic homeostasis, we generated a whole-body and a brown adipocyte-specific *P2rx5*-knockout mouse model. In mild cold exposure, *P2rx5*-deficiency resulted in reduced expression of thermogenic genes such as *Ucp1*, an observation that was confirmed in differentiated brown adipocytes in vitro. Nevertheless, indirect calorimetry studies indicated that P2X5 did not influence systemic energy expenditure in mice housed at different ambient temperatures. On the other hand, P2X5 influenced metabolic parameters, as shown by lower plasma cholesterol and free fatty acid levels observed in mice with brown adipocyte-specific *P2rx5*-deletion. Notably, under chow and in high-fat diet-fed obesogenic conditions, *P2rx5* deficiency in brown adipocytes resulted in an impaired glucose tolerance which was explained by lower glucose disposal into BAT. Overall, our data show that purinergic signalling via P2X5 is dispensable for energy expenditure but that it shapes expression of thermogenic genes and systemic glucose handling.

## 2. Results

### 2.1. P2rx5 Is Highly Expressed by Mature Brown Adipocytes

**Figure 1:**
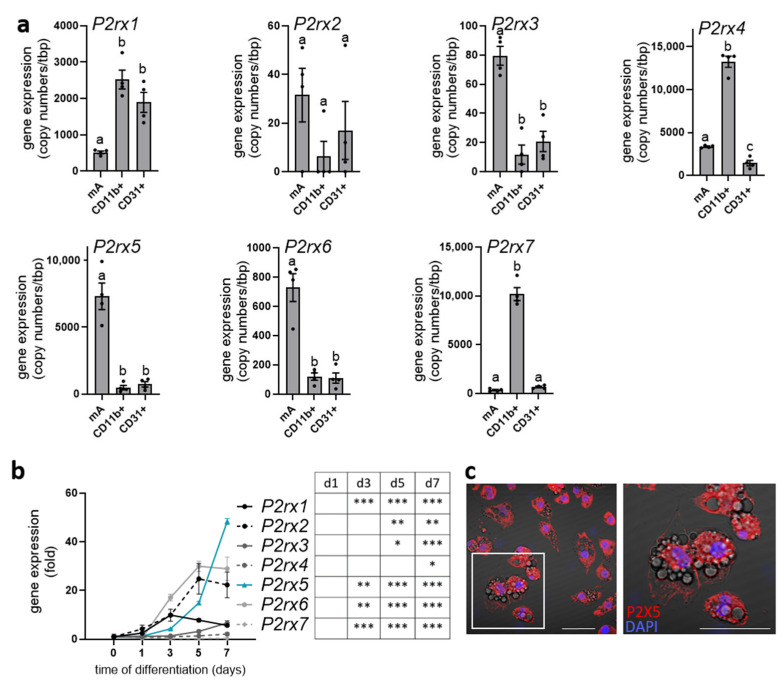
*P2rx5* is highly expressed by mature brown adipocytes. (**a**) Cell type-specific gene expression of the purinergic P2X receptors (*P2rx1*, *P2rx2*, *P2rx3*, *P2rx4*, *P2rx5*, *P2rx6*, *P2rx7*) in mature brown adipocytes (mA), CD11b+ myeloid cells, and CD31+ endothelial cells isolated from murine brown adipose tissue (BAT) using magnetic-activated cell sorting (MACS) (*n* = 4); (**b**) gene expression of *P2rx1*, *P2rx2*, *P2rx3*, *P2rx4*, *P2rx5*, *P2rx6*, and *P2rx7* during the differentiation of stromal-vascular cell (SVC)-derived brown adipocytes (*n* = 4); (**c**) P2X5 staining in primary brown adipocytes. Red, P2X5; blue, DAPI. Scale bar: 50 µm. Data are presented as mean values ± SEM. (**a**) A one-way ANOVA followed by Tukey’s multiple comparisons test comparing cell fractions with each other was performed. Different letters denote significant differences between cell fractions. (**b**) A one-way ANOVA followed by Dunnett’s multiple comparisons test comparing the different days of differentiation to day 0. In the table, significant differences are indicated by asterisks (* *p* < 0.05, ** *p* < 0.01, *** *p* < 0.001).

P2X5 was described as a cell surface marker for brown adipocytes, with its highest expression in brown adipose tissue (BAT) compared to other organs such as white adipose tissue, the heart, or the liver (Supplementary Figure S1) [27]. To further study the cell type-specific expression of all purinergic P2X receptors (*P2rx1*–*7*) in BAT, we isolated mature brown adipocytes (mA), tissue-resident myeloid cells including macrophages (CD11b+), and endothelial cells (CD31+) by magnetic-activated cell sorting (MACS^®^) (Figure 1a). Of note, *P2rx5* was predominantly expressed by mature brown adipocytes with a high copy number. Similarly, *P2rx3* and *P2rx6* were mainly detected in the brown adipocyte fraction but at a lower abundance, as indicated by much lower copy numbers. While *P2rx1* was expressed by myeloid and endothelial cells, *P2rx4* and *P2rx7* were marker genes for myeloid cells in the BAT. Moreover, *P2rx5* gene expression increased during the differentiation of BAT-derived stromal vascular cells to mature brown adipocytes and displayed the highest fold induction of all P2X receptors (Figure 1b). Next to gene expression, we detected endogenous P2X5 protein at the plasma membrane of terminally differentiated brown adipocytes (Figure 1c). In line with previous studies describing P2X5 as a brown adipocyte cell surface marker [27], these data confirm that *P2rx5* is predominantly expressed by mature brown adipocytes in BAT and is upregulated upon adipogenic differentiation.

### 2.2. P2rx5 Deficiency Resulted in Lower Expression of Thermogenic Markers in BAT

**Figure 2:**
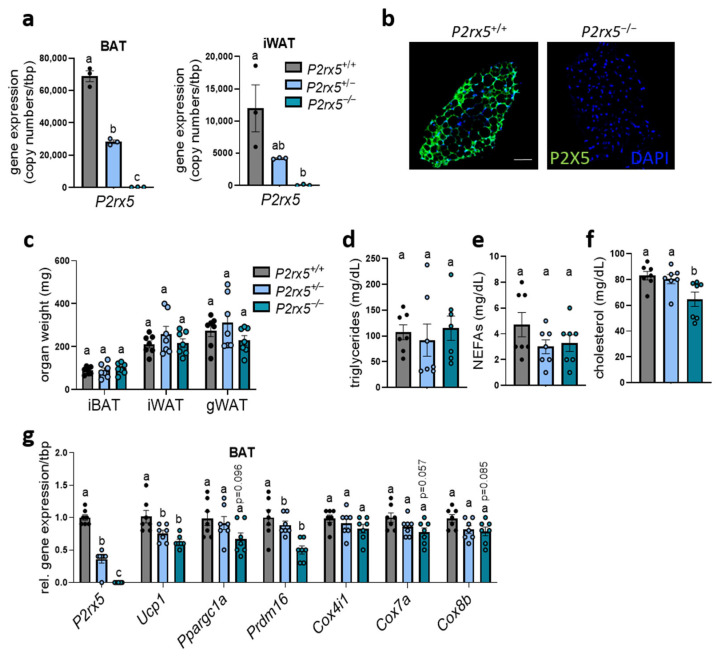
*P2rx5* deficiency resulted in lower expression of thermogenic markers in BAT. (**a**–**g**) *P2rx5* knockout mice (*P2rx5^−/−^*), heterozygous (*P2rx5*^+/−^), and controls (*P2rx5^+/+^*) were housed at 22 °C. (**a**) Gene expression of P2rx5 in BAT and inguinal white adipose tissue (iWAT) (*n* = 3). (**b**) Whole-mount staining of P2X5 in BAT of *P2rx5^−/−^* and *P2rx5^+/+^* mice. Green, P2X5; blue, DAPI. Scale bar: 50 µm. (**c**) Adipose tissue weights, (**d**) plasma triglycerides, (**e**) plasma non-esterified fatty acids (NEFAs), and (**f**) plasma cholesterol levels of *P2rx5* knockout mice (*P2rx5^−/−^*), heterozygous (*P2rx5*^+/−^), and controls (*P2rx5^+/+^*) (*n* = 7). (**g**) Gene expression of thermogenic marker genes in BAT of *P2rx5^−/−^, P2rx5*^+/−^, and *P2rx5^+/+^* mice housed at 22 °C (*n* = 7). Data are presented as mean values ± SEM. A one-way ANOVA followed by Tukey’s multiple comparisons test comparing wild type, heterozygous, and homozygous P2rx5 knockout mice was performed. Different letters denote significant differences.

To investigate the relevance of *P2rx5* for adaptive thermogenesis, we generated a whole-body-deficient *P2rx5* mouse model. Basal characteristics were determined in homozygous (*P2rx5^−/−^*), heterozygous (*P2rx5*^+/−^) knockout and wild type (*P2rx5*^+/+^) littermates housed at room temperature (22 °C), which is a mild cold exposure for mice. In BAT, we observed a high *P2rx5* expression in wild type mice, whereas heterozygous mice display a 50% reduction (Figure 2a). Next to gene expression, we confirmed the successful deletion of P2X5 on a protein level by immunohistological staining of BAT comparing wild type and homozygous *P2rx5* knockout mice (Figure 2b). A similar pattern, but with a lower expression level, was observed in WAT, which is most likely due to *P2rx5* expression by beige adipocytes. In these and other tissues, we showed a complete deletion of *P2rx5* in homozygous knockout mice (Figure 2a, Supplementary Figure S1). Under these conditions we detected similar adipose tissue weights (interscapular BAT, inguinal WAT, and gonadal WAT), plasma triglycerides, and non-esterified fatty acids, as well as lower cholesterol levels, when comparing the different groups (Figure 2c–f). In the BAT of *P2rx5*-knockout mice, gene expression of thermogenic marker genes was significantly lower (*Ucp1*, *Prdm16*) or showed a tendency (*Ppargc1a*, *Cox7a*, *Cox8b*) to be decreased compared to the control or heterozygous mice (Figure 2g).

**Figure 3:**
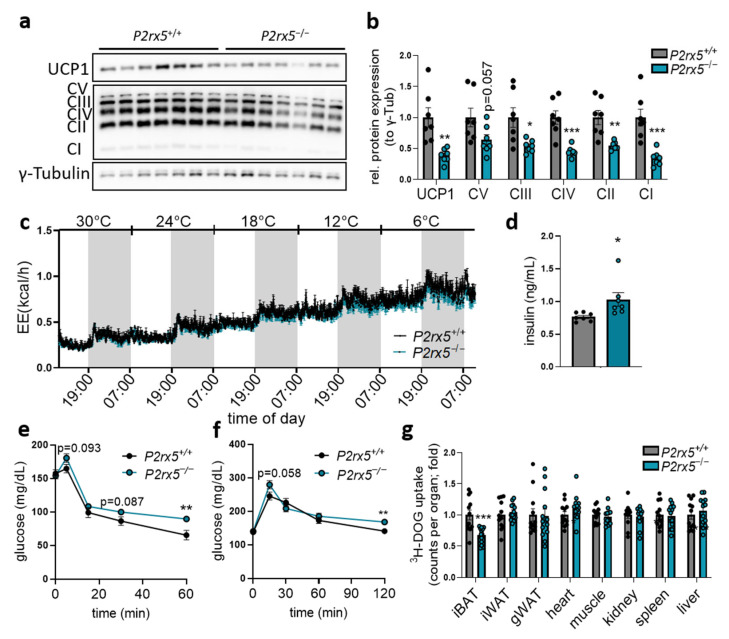
*P2rx5* deficiency improves glucose disposal by BAT. (**a**–**b**,**d**–**g**) *P2rx5* knockout mice (*P2rx5^−/−^*), and controls (*P2rx5^+/+^*) were housed at 22 °C. (**a**) Western blot analysis of UCP1, respiratory chain (OXPHOS) complexes, and g-Tubulin in BAT as well as (**b**) quantification of proteins shown in (**a**) (*n* = 7). (**c**) Energy expenditure in *P2rx5^+/+^* and *P2rx5^−/−^* mice housed at indicated environmental temperatures (30 °C, 24 °C, 18 °C, 12 °C, and 6 °C with a stepwise decrease in temperature every 24 h) in metabolic chambers (*n* = 6). (**d**) Plasma insulin levels of *P2rx5^+/+^* and *P2rx5^−/−^* mice (*n* = 7). (**e**) Blood glucose levels during insulin tolerance test (*n* = 12) and (**f**) blood glucose levels during oral glucose tolerance test (*n* = 12–13) of *P2rx5^+/+^* and *P2rx5^−/−^* mice. (**g**) Uptake of ^3^H-deoxyglucose (DOG) into interscapular BAT (iBAT), inguinal WAT (iWAT), gonadal WAT (gWAT), heart, muscle, kidney, spleen, and liver (*n* = 12–13). Data are presented as mean values ± SEM. * *p* < 0.05, ** *p* < 0.01, and *** *p* < 0.001 by Student’s *t* test.

In line with this, protein levels of UCP1 and the respiratory OXPHOS complexes were lower in the BAT of mice lacking *P2rx5* (Figure 3a,b). The importance of *P2rx5* for systemic energy expenditure during different ambient temperatures was directly investigated by housing *P2rx5*-knockout and control mice in an indirect calorimetry system. Here, the housing temperature was decreased stepwise (6 °C per day) starting at thermoneutrality (30 °C) down to 6 °C. Despite lower UCP1 expression, the energy expenditure, body core temperature, and body weights of *P2rx5*-knockout mice were comparable to wild type controls (Figure 3c, Supplementary Figure S2).

Next to its contribution to systemic energy expenditure, BAT has a capacity to internalize energy-rich substrates such as glucose [8]. As extracellular ATP released by pannexin 1 has been shown to regulate adipocyte glucose uptake [30], we determined a potential role of *P2rx5* for systemic glucose metabolism. Compared to control wild type littermates, basal plasma insulin levels were higher in *P2rx5^−/−^* mice (Figure 3d), which is in line with the impaired insulin sensitivity shown by higher glucose levels during insulin tolerance tests (Figure 3e). Moreover, global *P2rx5* deficiency resulted in a slightly impaired glucose tolerance (Figure 3f) that can be explained by lower glucose uptake specifically by BAT but not other organs, as shown by diminished uptake of the orally applied radioactive tracer ^3^H-deoxyglucose (Figure 3g). Overall, these findings indicate that *P2rx5* regulates the expression of thermogenic mediators such as UCP1 but is dispensable for whole-body energy expenditure in mice exposed to different ambient temperatures. Notably, *P2rx5* promotes glucose disposal by BAT and thereby it regulates systemic glucose homeostasis.

### 2.3. Brown Adipocyte-Specific P2rx5 Deletion Resulted in Lower UCP1 Expression and Impaired Glucose Tolerance

**Figure 4:**
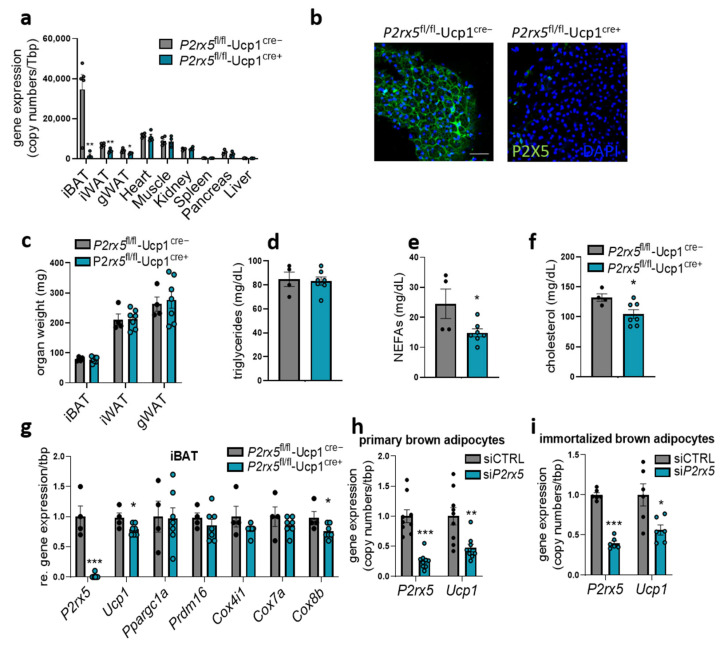
Brown adipocyte-specific *P2rx5* deletion resulted in lower Ucp1 expression. (**a**–**g**) Brown adipocyte-specific *P2rx5*-deficient mice (*P2rx5^fl/fl^*-Ucp1^Cre+^) and *P2rx5^fl/fl^*-Ucp1^Cre−^ mice were housed at 22 °C. (**a**) Gene expression of *P2rx5* in BAT, inguinal WAT (iWAT), gonadal WAT (gWAT), heart, muscle, kidney, spleen, pancreas, and liver of the *P2rx5^fl/fl^*-Ucp1^Cre^ model (*n* = 4–5). (**b**) Whole-mount staining of P2X5 in BAT of *P2rx5^fl/fl^*-Ucp1^Cre+^ and *P2rx5^fl/fl^*-Ucp1^Cre−^ mice. Green, P2X5; blue, DAPI. Scale bar: 50 µm. (**c**) Adipose tissue weights, (**d**) plasma triglycerides, (**e**) plasma non-esterified fatty acids (NEFAs), and (**f**) plasma cholesterol levels of *P2rx5^fl/fl^*-Ucp1^Cre+^ and *P2rx5^fl/fl^*-Ucp1^Cre−^ mice (*n* = 4–7). (**g**) BAT gene expression of thermogenic marker genes in *P2rx5^fl/fl^*-Ucp1^Cre+^ and *P2rx5^fl/fl^*-Ucp1^Cre−^ mice housed at 22 °C (*n* = 4–7). (**h**,**i**) siRNA-mediated knockdown of *P2rx5* was induced in primary stromal-vascular cell (SVC)-derived brown adipocytes and in immortalized brown adipocytes for 48 h. (**h**) Gene expression of *P2rx5* and *Ucp1* in primary SVC-derived (*n* = 9, knockdown 74%) or (**i**) immortalized pre-adipocytes (*n* = 6, knockdown 60%) differentiated to mature brown adipocytes. Data are presented as mean values ± SEM. * *p* < 0.05, ** *p* < 0.01, and *** *p* < 0.001 by Student’s *t* test.

To assess the cell type specific role of *P2rx5* expressed by brown adipocytes, we generated brown adipocyte-specific *P2rx5*-knockout mice (*P2rx5*^fl/fl^-Ucp1^Cre+^) as well as control littermates (*P2rx5*^fl/fl^-Ucp1^Cre−^). Successful deletion of *P2rx5* was detected in BAT, again verifying *P2rx5* to be predominantly expressed by the brown adipocytes but not other cell types in thermogenic adipose tissues (Figure 4a). Since Ucp1+ positive beige adipocytes are present in WAT depots upon thermogenic activation in mice housed below thermoneutrality, a lower expression of *P2rx5* was also detected in iWAT and gonadal WAT (gWAT) of *P2rx5*^fl/fl^-Ucp1^Cre+^ compared to *P2rx5*^fl/fl^-Ucp1^Cre−^ controls (Figure 4a). In various other organs, e.g., the heart, muscle, and the liver, *P2rx5* expression levels were unaffected (Figure 4a). In line with the mRNA expression data, efficient depletion of P2X5 in BAT could be demonstrated by immunohistochemistry (Figure 4b). Under these conditions, *P2rx5*^fl/fl^-Ucp1^Cre+^ and *P2rx5*^fl/fl^-Ucp1^Cre−^ mice display comparable adipose tissue weights as well as plasma triglyceride levels (Figure 4c,d). Plasma non-esterified fatty acid and cholesterol levels were decreased in *P2rx5*^fl/fl^-Ucp1^Cre+^ mice compared to controls (Figure 4e,f). Similarly to whole-body *P2rx5*-knockout mice, lack of *P2rx5* in brown adipocytes resulted in lower *Ucp1* expression (Figure 4g). To confirm this finding on a cellular level, we knocked-down *P2rx5* in differentiated primary and immortalized murine brown adipocytes in vitro using an siRNA-mediated approach. In line with the results obtained in vivo, *P2rx5* knockdown is associated with lower *Ucp1* expression (Figure 4h,i).

**Figure 5:**
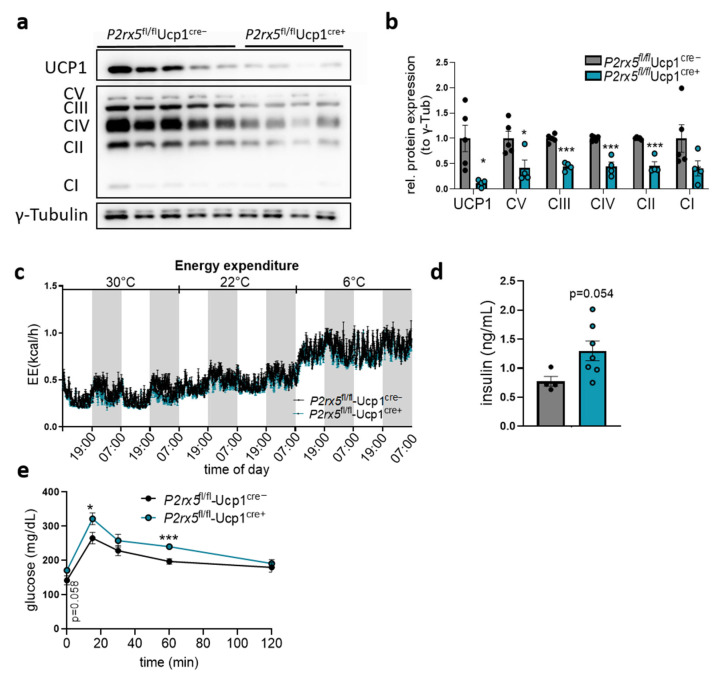
Brown adipocyte-specific *P2rx5* deletion impaired glucose tolerance. (**a**,**b**,**d**,**e**) Brown adipocyte specific *P2rx5*-deficient mice (*P2rx5^fl/fl^*-Ucp1^Cre+^) and *P2rx5^fl/fl^*-Ucp1^Cre−^ mice were housed at 22 °C. (**a**) Western blot analysis of BAT and (**b**) quantification of proteins shown in (**a**) (*n* = 4–5). (**c**) Energy expenditure in *P2rx5^fl/fl^*-Ucp1^Cre+^ and *P2rx5^fl/fl^*-Ucp1^Cre−^ mice housed at indicated environmental temperatures in metabolic chambers. Here, ambient temperature was decreased every other day starting at thermoneutrality (30 °C) to mild cold exposure (22 °C) and cold exposure (6 °C) (*n* = 3). (**d**) Plasma insulin levels in *P2rx5^fl/fl^*-Ucp1^Cre+^ and *P2rx5^fl/fl^*-Ucp1^Cre−^ mice. (**e**) Blood glucose levels during oral glucose tolerance tests in *P2rx5^fl/fl^*-Ucp1^Cre+^ and *P2rx5^fl/fl^*-Ucp1^Cre−^ mice (*n* = 6). Data are presented as mean values ± SEM. * *p* < 0.05, and *** *p* < 0.001 by Student’s *t* test.

Protein levels of UCP1 and the respiratory OXPHOS complexes were decreased in the BAT of mice lacking *P2rx5* specifically in brown adipocytes (Figure 5a,b). Similarly to the whole-body knockout, indirect calorimetry studies indicated comparable energy expenditure as well as similar body core temperature and body weights of *P2rx5*^fl/fl^-Ucp1^Cre+^ and *P2rx5*^fl/fl^-Ucp1^Cre−^ mice at different temperature conditions (Figure 5c, Supplementary Figure S3). In line with the global knockouts, we observed a trend for higher basal plasma insulin (Figure 5d) and an impaired blood glucose clearance in *P2rx5*^fl/fl^-Ucp1^Cre+^ mice compared to *P2rx5*^fl/fl^-*Ucp1^Cre−^* mice (Figure 5e). Altogether, these data suggest that *P2rx5* expressed in brown adipocytes regulates Ucp1 expression and influences systemic glucose metabolism.

### 2.4. Brown Adipocyte-Specific P2rx5 Deficiency Impaired Glucose Tolerance and Uptake into BAT of Diet-Induced Obese Mice

**Figure 6:**
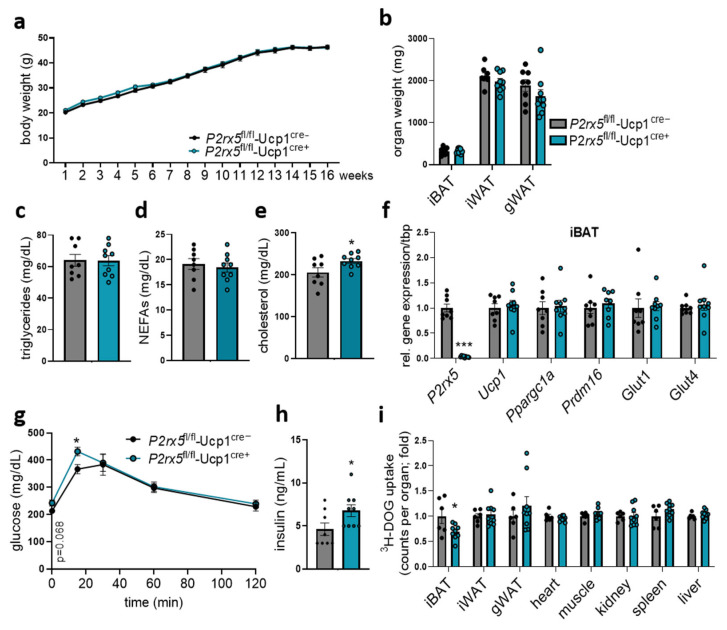
Brown adipocyte-specific *P2rx5* deficiency impaired glucose tolerance and uptake into the BAT of diet-induced obese mice. (**a**–**i**) Brown adipocyte-specific *P2rx5* deficient mice (*P2rx5*^fl/fl^-Ucp1^Cre+^) and *P2rx5*^fl/fl^-Ucp1^Cre−^ mice were fed a high-fat diet (HFD) for 16 weeks. (**a**) Body weights of *P2rx5*^fl/fl^-Ucp1^Cre+^ and *P2rx5*^fl/fl^-Ucp1^Cre−^ mice during the HFD feeding period (*n* = 9–10). (**b**) Adipose tissue weights, (**c**) plasma triglycerides, (**d**) plasma non-esterified fatty acids (NEFAs), and (**e**) plasma cholesterol levels of *P2rx5*^fl/fl^-Ucp1^Cre+^ and *P2rx5*^fl/fl^-Ucp1^Cre−^ mice (*n* = 8–9). (**f**) BAT gene expression (*n* = 8–9). (**g**) Blood glucose levels during the oral glucose tolerance test (OGTT) (*n* = 8–9). (**h**) Plasma insulin levels of *P2rx5*^fl/fl^-Ucp1^Cre+^ and *P2rx5*^fl/fl^-Ucp1^Cre−^ mice (*n* = 8–9). (**i**) Uptake of ^3^H-deoxyglucose (DOG) per total organ into interscapular BAT (iBAT), inguinal WAT (iWAT), gonadal WAT (gWAT), heart, muscle, kidney, spleen, and liver (*n* = 6–9). Data are presented as mean values ± SEM. * *p* < 0.05 by Student’s *t* test.

In obesity, insulin-resistant adipose tissue is associated with defective systemic glucose handling. To investigate glucose metabolism under obesogenic conditions, we challenged *P2rx5*^fl/fl^-Ucp1^Cre+^ mice and littermate controls by feeding a high-fat diet (HFD) to induce diet-induced obesity. After 16 weeks of HFD feeding, *P2rx5*^fl/fl^-Ucp1^Cre+^ mice and controls had similar body weights and organ weights, as well as plasma triglyceride and non-esterified fatty acid levels (Figure 6a–d). Plasma cholesterol levels were slightly elevated in mice lacking *P2rx5* in brown adipocytes (Figure 6e). Moreover, gene expression of thermogenic markers in BAT was unaffected in *P2rx5*^fl/fl^-Ucp1^Cre+^ mice compared to *P2rx5*^fl/fl^-Ucp1^Cre−^ mice (Figure 6f). In line with impaired blood glucose tolerance in conditions of regular diet feeding, obese mice with brown adipocyte-specific *P2rx5* deficiency had higher blood glucose levels at basal and 15 min after oral glucose gavage (Figure 6g), as well as elevated plasma insulin levels (Figure 6h). To quantify the organ-specific glucose uptake, we traced the glucose gavage solution with radiolabeled ^3^H-deoxyglucose. Remarkably, elevated systemic glucose levels can be explained by reduced ^3^H-deoxyglucose disposal by the BAT of mice lacking *P2rx5* in brown adipocytes (Figure 6i). Taken together, these data indicate that in brown adipocytes *P2rx5* shapes systemic glucose handling in both basal and obese conditions.

## 3. Discussion

Norepinephrine released by sympathetic nerves innervating the BAT induces heat production via adrenergic stimulation. The subsequent cAMP-mediated signalling cascades orchestrate the efficient handling of nutrients including the liberation of fatty acids from lipid droplets and the gene expression of thermogenic genes [3]. However, additional stimulatory but also inhibitory signalling pathways regulated by hormones and lipokines have been shown to modulate thermogenic responses in BAT and WAT. For instance, the adipocyte-derived released C-terminal fragment of *Slit2* induces a thermogenic programme through the protein kinase A signalling pathway, enhancing energy expenditure and glucose disposal in vivo [31]. Also, lipids with signalling properties, such as 12,13-dihydroxy-9Z-octadecenoic acid (12,13-diHOME), can be released by adipocytes in response to cold exposure to mediate uptake of energy-rich substrates including glucose and fatty acids [32]. Next to proteins and lipokines, adenine nucleotides have also been suggested to shape intercellular crosstalk and thermogenic function [33]. It is known that the levels of extracellular ATP increase in response to BAT stimulation, e.g., by electric field stimulation [18]. The subsequent hydrolysis by several ectoenzymes results in the formation of ADP, AMP, adenosine, and inosine. The latter have been intensively studied in adipose tissues, showing that both adenosine and inosine induce browning and activate thermogenesis via A2A- and A2B-receptor signalling [18,34,35]. In addition, adenosine generated by the ectoenzyme CD73 can be internalized and converted to AMP by intracellular adenosine kinase, shaping AMP kinase activity and thus endogenous lipid synthesis in adipocytes [36]. While these pathways focused on the degradation products of ATP, the potential relevance of direct ATP sensing by thermogenic adipocytes is less well understood. In this context it is of note that the intensively studied ATP receptor P2X7 is dispensable for energy expenditure under conditions of both diet-induced and cold-stimulated thermogenesis [26]. However, P2X7 is primarily expressed by immune cells present in WAT and BAT, while another member of this family, P2X5, has been identified as a cell surface marker of brown adipocytes [27]. In the current study, we confirmed that *P2rx5* is highly abundant in mature brown adipocytes. Based on metabolic studies using whole-body and brown adipocyte-specific *P2rx5* knockout mice, we found that P2X5 modulates thermogenic marker expression in BAT but is dispensable for systemic energy expenditure. Interestingly, global and brown adipocyte-specific *P2rx5* deficiency is associated with impaired glucose tolerance in both lean and diet-induced obese mice, most likely due to it regulating glucose disposal into BAT depots. In a recent report studying global P2X5 knockout mice, an anti-obesity effect was described in response to P2X5 agonism using a stabilized ATP analogue in mice which was explained by enhanced BAT recruitment [37]. Although lower UCP1 levels and OXPHOS proteins in the BAT of global and brown adipocyte-specific *P2rx5* deficient mice were detected in the current study, whole-body energy expenditure and body weight gain were similar when comparing the different genotypes. These discrepancies might be explained by different experimental study designs, as we did not administer pharmacological doses of ATP. Moreover, we show that P2X5 modulates BAT glucose uptake, which is associated with systemic glucose homeostasis. In light of the lower UCP1 expression levels, we cannot rule out the possibility that lower glucose uptake is also associated with impaired BAT-mediated energy expenditure. However, this mild effect may be too small to be detected in whole-body energy expenditure, which was determined by indirect calorimetry. Functioning as an ion channel, P2X5 has the potential to shuttle different ions between extra- and intracellular space and to evoke ion-driven signals [38]. Other ion channels have also been suggested to influence brown adipocyte thermogenesis by altering membrane potential during sympathetic activation. For instance, the potassium two-pore domain channel subfamily K member 3 (KCNK3) expressed by adipocytes limits Ca^2+^ influx by antagonizing membrane depolarization in response to norepinephrine [39]. Consequently, Ca^2+^ impulses needed for efficient activation of adenylate cyclase-dependent cAMP generation are dampened, lowering adaptive thermogenesis [39]. On the other hand, calcium-permeable channels of the transient receptor potential (TRP) ion channel family are described to induce thermogenic gene expression and mediating adipocyte thermogenesis [40]. For instance, the TRP vanilloid 2 (TRPV2) acts synergistically with beta-adrenergic signalling by inducing Ca^2+^ influx [41]. Furthermore, the depletion of the non-voltage-activated Ca^2+^ channel TRP melastatin 2 (TRPM2) resulted in the lower expression of thermogenic genes such as *Pgc1α* and *Ucp1* as well as diminished energy expenditure in response to cold exposure and the beta-3-adrenergic agonist CL316,243 [42]. In a similar manner, the observed decrease in UCP1 expression and impaired glucose disposal into BAT of *P2rx5*-deficient mice could be explained by lower ATP-induced Ca^2+^ influx and the subsequent dampening of cAMP-dependent signalling. In the current study, the lower expression of thermogenic markers does not impact whole-body energy expenditure and thermogenic capacity, which suggest compensatory mechanisms by other P2X receptors and/or TRP channels. In the human context it is of note that a single nucleotide polymorphism (SNP) in the 3′ splice site of *P2rx5* exon 10 is present in almost the entire European population but not in African populations [43]. This SNP results in a truncated dysfunctional protein [44], questioning from a European perspective to what extent P2X5 regulates human physiology. On the other hand, recent studies show that human populations that lived in extreme cold conditions have a number of genetic variants, e.g., in UCP1, tribbles pseudo-kinase 2 or beta-3-adrenergic receptor, that are associated with leanness and higher basal BAT thermogenesis [45]. Future studies are needed to investigate whether expression of the functional human P2X5 protein would result in reduced BAT activity and if it may explain the lower basal metabolic rates described for populations with African ancestry.

In conclusion, we report here that extracellular ATP could directly modulate thermogenic gene expression and systemic glucose metabolism via P2X5 expressed by brown adipocytes. In whole-body and brown adipocyte-specific knockout mice, P2X5 has no effect on energy expenditure.

## 4. Materials and Methods

### 4.1. Mice

All experiments were approved by the Behörde für Gesundheit und Verbraucherschutz, Hamburg (N082/2020). Mice were housed in the animal facility of the UKE at a 12 h light/12 h dark cycle with access to food and water ad libitum. Wild type mice (C57BL/6) were purchased from Charles River or bred in-house. *P2rx5* knockout mice (C57BL/6N-*P2rx5* ^<tm1a(EUCOMM)Hmgu>^/Ieg) were obtained from the European Mouse Mutant Archive (EMMA), Institute of Experimental Genetics, Helmholtz Zentrum, München (EMMA strain ID: EM:09185). To generate P2rx5^tm1c^ mice with a floxed exon 2 in the *P2rx5* gene, *P2rx5* ^<tm1a(EUCOMM)Hmgu>^/Ieg mice were bred with flippase (Flp)-expressing mice, deleting the FRT-flanked LacZ cassette. For the generation of a brown adipocyte-specific *P2rx5* knockout mouse model, these mice were then crossed with B6.FVB-Tg(Ucp1-Cre)1Evdr mice that were expressing the Cre-recombinase under the control of the UCP1 promotor (Jackson Laboratories stock #024670, Bar Harbor, ME, USA). For high-fat diet (HFD) feeding, the mice were fed for 16 weeks (D14010701; ResearchDiets, Inc., New Brunswick, NJ, USA). For organ harvest and blood collection, the mice were anesthetized by ketamine (180 mg/kg) and xylazine (24 mg/kg) injection after 4 h fasting.

### 4.2. Isolation of Adipocytes, Tissue-Resident Macrophages and Endothelial Cells

Isolation of cell type fractions from interscapular BAT was performed by magnetic-activated cell sorting (MACS^®^), as described previously [36]. Briefly, a pool of BAT derived from 4 mice was digested for 45 min using a buffer containing 1.5 U/mL collagenase D and 2.4 U/mL dispase II. The obtained cell suspension was passed through a 100 µm cell strainer and pelleted by centrifugation (5 min, 600× *g*). To harvest mature brown adipocytes (mA), the floating fraction was collected. The pellet was resuspended in PBS-based buffer containing 2 mM EDTA, 0.5% BSA, and 2 mM glucose. The sample was filtered through a 40 µm cell strainer, centrifuged (5 min, 600× *g*), and the remaining cell pellet resuspended. To isolate the macrophage fraction, the sample was incubated with CD11b microbeads (Miltenyi, 10 µL beads/10^7^ cells, Auburn, CA, USA) for 15 min. The CD11b+-fraction was separated using magnetic LS columns (Miltenyi). For endothelial cell isolation, remaining flow through was incubated with CD31 microbeads (Miltenyi). The obtained cell pellets of each fraction were dissolved in TRIzol^®^ for RNA isolation.

### 4.3. Gene Expression Analysis

For RNA isolation from whole-tissue samples, BAT cell fractions, or adipocyte cell cultures, TRIzol^®^ reagent and a NucleoSpin RNAII kit (Macherey & Nagel, Düren, Germany) were used according to the manufacturer’s instructions. A NanoPhotometer^®^ N60 spectrophotometer (IMPLEN, Munich, Germany) was used to determine RNA concentrations. Afterwards, 400 ng of RNA was transcribed into cDNA (High-Capacity cDNA Reverse Transcription Kit, Applied Biosystems, Waltham, MA, USA) following the company’s protocol. Quantitative real-time RT-PCR was performed on a QuantStudio 5 Real-Time-PCR System (ThermoFischer Scientific, Waltham, MA, USA). For this purpose the following TaqMan^®^ Assay-on-Demand primer sets were used: mCox4i1 (Mm00438289_g1), mCox7a (Mm00438297_g1), mCox8b (Mm00432648_m1), mGlut1 (Mm00441480_m1), mGlut4 (Mm01245502_m1), mP2rx1 (Mm00435460_m1), mP2rx2 (Mm00462952_m1), mP2rx3 (Mm00523699_m1), mP2rx4 (Mm00501787_m1), mP2rx5 (Mm00473677_m1/Mm01242356_m1), mP2rx6 (Mm00440591_m1), mP2rx7 (Mm01199500_m1), mPpargc1a (Mm00447183_m1), mPrdm16 (Mm00712556_m1), mTbp (Mm00446973_m1), and mUcp1 (Mm00494069_m1). Gene expression data obtained by qRT-PCR was either shown as copy number or as a relative comparison of the different groups. Copy numbers were calculated according to standard equations (copy number = 10^ct-intercept/slope^) using qPCR device-specific constants. The obtained copy numbers were displayed as a normalized virtual quantity by dividing them by the housekeeper’s copy number and multiplying by a factor of 10^4^. Relative mRNA expression was normalized to mTbp (TATA-box binding protein) as housekeeper and calculated via the ΔΔCT method. The *P2rx5* mRNA levels of the global knockout can be considered to be neglectable. However, the TaqMan probe used for analysis of the global knockout detected *P2rx5* expression with a high CT value. This can be explained as the TaqMan probe detected *P2rx5* at exon10/11, which suggests that an unstable *P2rx5* mRNA lacking exon 2 is expressed at a very low level.

### 4.4. Glucose Uptake Studies

For the oral glucose tolerance test (OGTT), mice received a glucose gavage containing 2 mg/g body weight glucose. To determine glucose uptake into organs, the gavage solution was traced with radiolabelled 3H-deoxyglucose (1.7 kBq/g body weight). At different time points (0, 15, 30, 60, and 120 min) after gavage, blood glucose concentrations were measured in blood taken from the tail vein using AccuCheck Aviva glucose sticks (Roche, Basel, Switzerland). After OGTT, mice were anesthetized and transcardially perfused with phosphate-buffered saline containing 10 U/mL heparin. Harvested tissues were dissolved in SolvableTM and radioactivities were determined using liquid scintillation counting (Tricarb, Perkin Elmer, Shelton, CT, USA).

### 4.5. Insulin Tolerance Test

For the insulin tolerance test (ITT), mice received an insulin injection (i.p.; 1 U/kg body weight) after 4 h of fasting. Blood glucose levels were determined in tail blood at different time points (0, 5, 15, 30, and 60 min) after injection.

### 4.6. Indirect Calorimetry

For indirect calorimetry, mice were kept in metabolic cages (Promethion^®^, Sable Systems, Las Vegas, NV, USA) and exposed to various ambient temperatures (as indicated). Here, the housing temperature was either decreased stepwise (6 °C per day) starting at thermoneutrality (30 °C) down to 6 °C or decreased every second day from thermoneutrality (30 °C) to 22 °C and 6 °C. Oxygen consumption and carbon dioxide production were monitored continuously. Analysis of the obtained data files was performed according to the manufacturer (Sable Systems) using the Macro interpreter software (v2.41).

### 4.7. Plasma Parameters

Lipid levels in plasma samples (cholesterol, triacylglycerides, and non-esterified fatty acids) were determined by enzymatic-colorimetric assays (cholesterol/triglyceride: Roche; non-esterified fatty acids: FUJIFILM Wako Chemicals Europe GMBH, Neuss, Germany) according to the manufacturer’s instructions. Insulin levels in the plasma samples were detected by ELISA (Crystal Chem, Elk Grove, IL, USA) according to the manufacturer’s protocol.

### 4.8. Cell Culture and siRNA-Mediated Knockdown

Primary brown adipocytes were obtained by isolation of the stromal-vascular fractions (SVF) from the BAT of wild type mice (C57BL/6J, male, age 4–6 weeks), as described previously [36]. For differentiation to mature adipocytes, SVF-derived cells were cultured in DMEM/high glucose GlutaMAX (Gibco, Grand Island, NY, USA) supplemented with 10% neonatal calf serum, 1% penicillin and streptomycin, 1% antibiotic-antimycotic, 2.4 nM of insulin, and 1 µM of rosiglitazone (Cayman Chemicals, Ann Arbor, MI, USA).

For immortalized brown adipocyte cell culture, precursor cells (WT1; [46]) were cultured in DMEM/high glucose supplemented with 10% FBS (Gibco) and 1% penicillin and streptomycin (maintenance media). Differentiation was induced two days post confluency by changing to differentiation media (DMEM/high glucose supplemented with 10% FBS (Gibco), 1% penicillin and streptomycin, 10 µg/mL insulin (Sigma, St. Louis, MO, USA), 1 µM IBMX (Sigma), 1 µM dexamethasone (Sigma), 1 µM rosiglitazone (Cayman Chemicals), 1 nM T3 (Sigma), and 50 µM indomethacine (Sigma)). At day 2 of differentiation, culture media was changed to maintenance media containing 10 µg/mL insulin, 1 µM rosiglitazone, and 1 nM T3. At day 4 of differentiation, cells received maintenance media supplemented with 10 µg/mL insulin. Media was changed to a regular maintenance medium at day 6.

siRNA-mediated knockdown of P2rx5 was induced by incubating primary or immortalized brown adipocytes with either anti-P2rx5 Silencer^®^ Select siRNA (Assay ID: s206955; ThermoFisher Scientific) or Silencer^®^ Negative Control #1 siRNA (AM4611, Invitrogen). For the siRNA transfection, Lipofectamine^®^ RNAiMAX Reagent (13778, ThermoFisher) was used according to manufacturer’s instructions. In brief, siRNA stocks were diluted in OptiMEM^®^ Medium (Gibco) to obtain a final concentration of 100 pmol/mL. Lipofectamine^®^ RNAiMAX Reagent was diluted in OptiMEM^®^ Medium, as described by the company. Diluted siRNA was added to the diluted Lipofectamine^®^ RNAiMAX Reagent in a 1:1 ratio. After 5 min of incubation, the mix was added dropwise to the cells. Cells were harvested in Trizol^®^ after 48 h of siRNA-mediated knockdown.

### 4.9. Western Blotting

Lysis of snap-frozen brown adipose tissue was performed in a RIPA buffer containing protease inhibitors (Complete Mini protease inhibitor cocktail; Roche), 0,1% SDS, and phosphatase inhibitors (1% phosphatase inhibitor A + B; bimake) using the TissueLyser (Qiagen, Hilden, Germany). Protein samples (20 µg) were separated on 10% and 12.5% acrylamide SDS-PAGE gels. After Western blot transfer, the nitrocellulose membranes were blocked in 5-milk in TBS-T (20 mM Tris, 150 mM NaCl, 0.1% (*v*/*v*) Tween 20) for 1 h. Incubation with primary antibodies (diluted in 5% BSA in TBS-T) was performed overnight at 4 °C. Afterwards, the membranes were washed with TBS-T and incubated with secondary HRP-conjugated antibodies (1:5000) for 1 h. Used primary antibodies were as follows: rabbit-anti-gTubulin (1:2000; Abcam #ab179503, Cambridge, UK; RRID: N/A), mouse-anti-OXPHOS (1:20,000; Abcam #ab110413; RRID:AB_2629281), and mouse-anti UCP1 (1:2500; R&D MAB6158; RRID:AB_10572490). Used secondary antibodies were as follows: Peroxidase-conjugated AffiniPure goat-anti-mouse IgG (H + L) (1:5000; Jackson ImmunoResearch #115-035-003, Philadelphia, PA, USA; RRID:AB_10015289) and Peroxidase-conjugated AffiniPure goat-anti-rabbit IgG (H + L) (1:5000; Jackson ImmunoResearch #111-035-144; RRID:AB_ 2307391). Enhanced chemiluminescence was detected using an Amersham Imager 600 (GE Healthcare, Chicago, IL, USA). For Signal quantification, ImageStudio Lite 5.2 (Licor, Lincoln, NE, USA; RRID:SCR_013715) was used.

### 4.10. Immunofluorescence Staining

Primary brown adipocytes were fixed with 4% paraformaldehyde and permeabilized with 0.2% TritonX100 in PBS. Afterwards, cells were blocked with 3% BSA in PBS and incubated with a polyclonal serum of rats immunized with murine P2X5 diluted in blocking buffer overnight at 4 °C. Secondary antibody incubation was performed for 2 h at room temperature using Cy5-donkey-anti-rat (1:300; Jackson ImmunoResearch Labs #712-175-153, RRID:AB_2340672). DAPI staining was performed during the subsequent mounting process by using ROTI^®^Mount FluorCare DAPI (ROTH, Karlsruhe, Germany).

For whole-mount immunofluorescent staining, the BAT was fixed with 4% paraformaldehyde and cut into small pieces. The tissue was washed three times with PBS, then immersed in 5% glycine for 45 min to quench autofluorescence. After blocking and permeabilization for 2 h using 3% BSA, 0.3% Triton X100, and 0.1% sodium citrate in PBS, the tissues were incubated with the above-mentioned polyclonal serum of rats immunized with murine P2X5 diluted in blocking buffer over night at 4 °C. Subsequently, the tissue pieces were washed three times with PBS and incubated with either a secondary Alexa Fluor^®^ 488-donkey-anti-rat or Cy3-donkey-anti-rat (1:300; Jackson ImmunoResearch Labs #712-546-153/#712-165-153, RRID:AB_2340686/RRID:AB_2340667) for 2 h at room temperature. Afterwards, tissues were washed three times with PBS and nuclei were stained using DAPI. For imaging, BAT pieces were transferred to dishes with a glass bottom and visualized using a NikonA1 Ti confocal microscope (Tokyo, Japan).

### 4.11. Quantification and Statistics

Data are expressed as mean ± S.E.M. Two groups were compared by an unpaired, two-tailed Student’s *t*-test, and more than two groups were compared by ANOVA. The statistical parameters such as *p* values and numbers of replicates are presented in the figure legends. Statistical analysis and presentation of data were conducted using Microsoft Excel 2016 (RRID:SCR_016137) and GraphPadPrism 9.0 (RRID:SCR_002798).

## Data Availability

Data is contained within the article and Supplementary Materials.

## Supplementary Materials

The following supporting information can be downloaded at: https://www.mdpi.com/article/10.3390/ijms26136474/s1.

## Abbreviations

The following abbreviations are used in this manuscript:

ATP	Adenosine triphosphate
BAT	Brown adipose tissue
DOG	Deoxyglucose
WAT	White adipose tissue
SVF	Stromal-vascular fractions
UCP1	Uncoupling protein 1
