# Supplementary material for: The Purinergic Receptor P2X5 Modulates Glucose Metabolism and Expression of Thermogenic Genes in Brown Adipose Tissue
Source: Int J Mol Sci. 2025 Jul 4;26(13):6474. doi: 10.3390/ijms26136474 (PMC12249994; doi:10.3390/ijms26136474)
Supplement: Supplementary file 1 [file ijms-26-06474-s001.zip › ijms-3657795-supplementary.pdf]

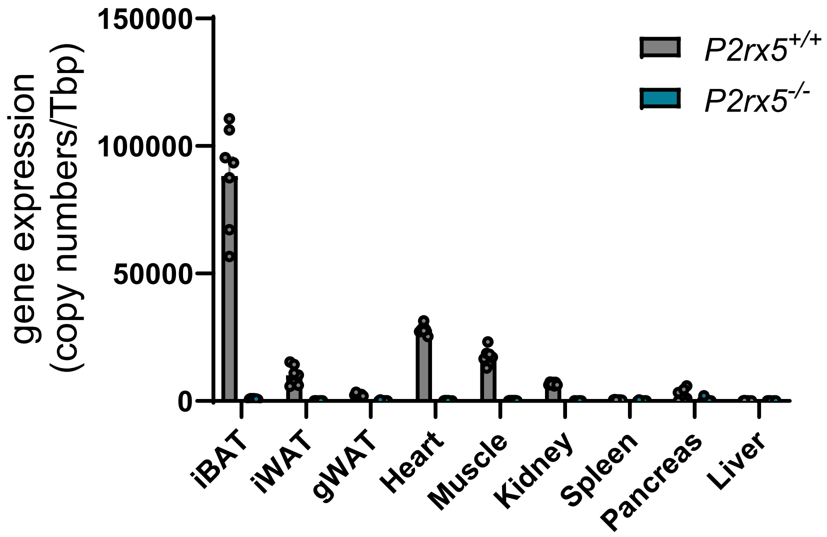

Supplementary Figure S1. Gene expression of *P2rx5* in various tissues from *P2rx5*$^{-/-}$ knockout mice and wild type (*P2rx5*$^{+/+}$) littermate controls (n=7). Data are presented as mean values ± SEM.

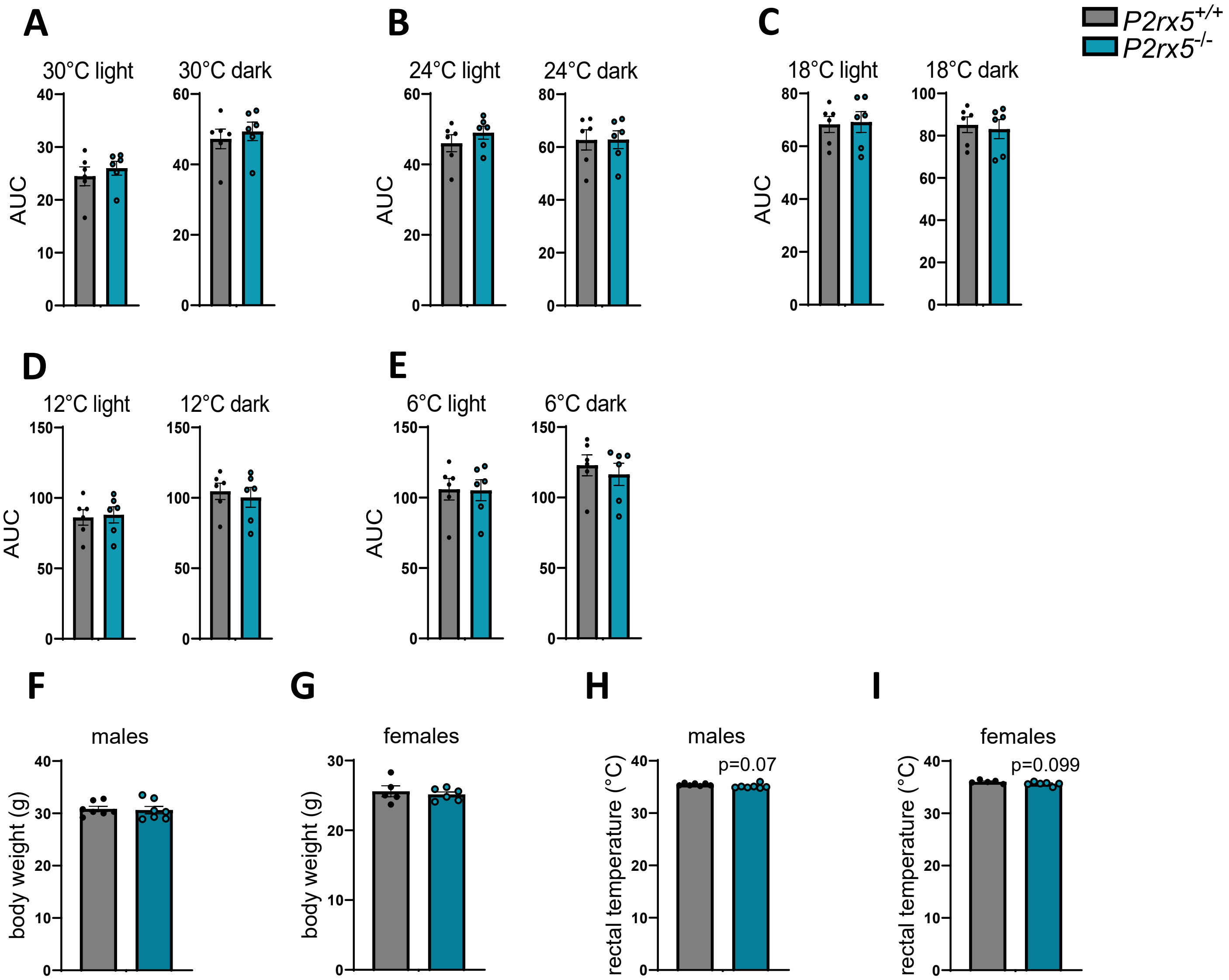

Supplementary Figure S2. *P2rx5* deficiency resulted in lower expression of thermogenic markers in BAT. **(A-E)** Area under the curve (AUC) quantification of the energy expenditure in homozygous (*P2rx5*$^{-/-}$) knockout mice and wild type (*P2rx5*$^{+/+}$) littermate controls housed at indicated environmental temperatures in metabolic chambers (n=6). **(F+G)** Body weights and **(H+I)** rectal temperature of male (n=7) and female (n=5-6) *P2rx5*$^{-/-}$ knockout mice and *P2rx5*$^{+/+}$ controls. Data are presented as mean values ± SEM.

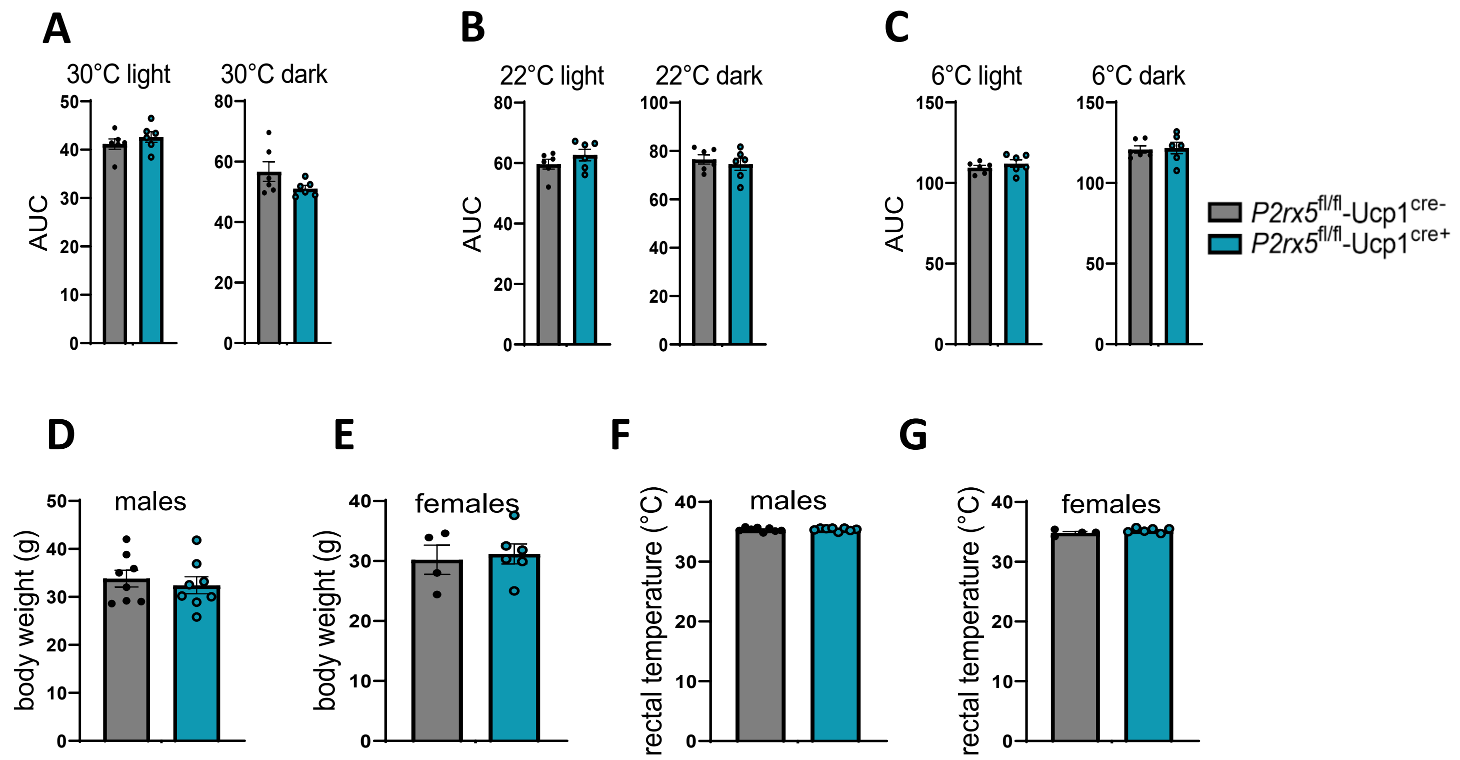

Supplementary Figure S3. Brown adipocyte specific *P2rx5* deletion resulted in lower Ucp1 expression and impaired glucose tolerance. **(A-C)** Area under the curve (AUC) quantification of the Energy expenditure in *P2rx5*$^{fl/fl}$-Ucp1^{Cre+} and *P2rx5*$^{fl/fl}$-Ucp1^{Cre-} mice housed at indicated environmental temperatures in metabolic chambers. **(D+E)** Body weights and **(F+G)** rectal temperature of male (n=8) and female (n=4-6) *P2rx5*$^{fl/fl}$-Ucp1^{Cre+} and *P2rx5*$^{fl/fl}$-Ucp1^{Cre-} mice. Data are presented as mean values ± SEM.
